# Complementary and Alternative Medicine Use in Amyotrophic Lateral Sclerosis Cases in South Korea

**DOI:** 10.1155/2019/4217057

**Published:** 2019-07-25

**Authors:** Sungha Kim, Sujeong Mun, Jeonghwan Park, Sunmi Choi, Sanghun Lee, Sungchul Kim

**Affiliations:** ^1^Clinical Medicine Division, Korea Institute of Oriental Medicine, 1672 Yuseong-daero, Yuseong-gu, Daejeon 34054, Republic of Korea; ^2^Future Medicine Division, Korea Institute of Oriental Medicine, 1672 Yuseong-daero, Yuseong-gu, Daejeon 34054, Republic of Korea; ^3^Department of Acupuncture & Moxibustion, Wonkwang University Gwangju Medical Hospital, Gwangju 61729, Republic of Korea

## Abstract

Patients with amyotrophic lateral sclerosis (ALS) sometimes consider complementary and alternative medicine (CAM) because of ineffective treatment. This study investigated the prevalence and utilization pattern of CAM among patients with ALS in South Korea. Participants were recruited through homecare services for mechanical ventilation in South Korea. This study comprised a face-to-face cross-sectional survey with staff members available to address any queries. Fifty-five participants were included; all had used >1 CAM treatment option for ALS symptoms. Dietary treatments were most common, followed by functional food and massages. Most participants had obtained relevant information from family members or friends. The main reason for CAM use was an expectation that symptoms will improve with CAM; most patients were unsure of the effects. CAM use was previously discontinued by the majority of patients because of unsatisfactory effects. The mean expenditure on CAM was 288,385.28 ± 685,265.14 won per month, and the mean duration of CAM use was 11.54 ± 20.09 months. The results indicate that there is a high prevalence of CAM use among ALS patients. Healthcare providers should inquire about CAM use and openly provide accurate CAM information. Further evidence of CAM efficacy is required, as is specific guidance for consulting ALS patients regarding CAM.

## 1. Introduction

The use of complementary and alternative medicine (CAM) is growing and will continue to affect healthcare delivery in the foreseeable future [1, 2]. Over 100 million Europeans are currently CAM users, with one-fifth of them using it exclusively and the same number preferring health care including CAM. There are numerous users of CAM in Africa, Asia, Australia, and North America [2].

More specifically, patients with amyotrophic lateral sclerosis (ALS) consider CAM because of the lack of effective conventional drugs [3, 4]. They use CAM with vague hopes and beliefs of improvement, despite knowing that it is considered an unorthodox method with no definite evidence of efficacy [5, 6]. It has been reported that 54% of ALS patients have tried CAM methods including acupuncture and homeopathy in a survey conducted by the German Association for Neuromuscular Diseases [7], while 42% used herbal supplements or other alternative therapies (15%) at the Utah Motor Neuron Disease Clinic in the United States [8]. In Shanghai, China, a higher number of ALS patients (99%) reported the use of at least 1 CAM treatment [9]. However, there are few studies focusing on the utilization of CAM in ALS cases [3], and there have been no published attempts to investigate the prevalence of CAM in South Korea. Therefore, this study investigated CAM use in patients with ALS in South Korea to determine the prevalence, motives, information sources, adverse effects, and cost of CAM.

## 2. Materials and Methods

### 2.1. Sample

This study comprised a face-to-face cross-sectional survey. The participants were recruited through home care services for mechanical ventilation. Because of the paralysis of respiratory muscles, all ALS patients need ventilators in the late stages. The South Korean government supports ventilator rental services for patients with ALS, and the ventilators are managed by home care services. Sampling through a home care service was a feasible method to recruit a representative population with ALS.

The purpose of the study was described, and written consent was obtained; then, willing participants took part in the survey during the visit with staff available to address any queries. All participants were informed that participation was voluntary.

Specific inclusion criteria for patients with ALS included the ability to express oneself or communicate through caregivers. Patients with stroke, dementia, or any cognitive disorders were excluded.

### 2.2. Questionnaire

A structured questionnaire for patients with ALS was developed and revised by 3 Korean medicine doctors, based on previous qualitative research [6] and the Home Remedy questionnaire developed by the Korean Institute of Oriental Medicine [10, 11]. In the questionnaire, CAM was defined as either self-treatment, or a recommendation, or the administration of a procedure by a noninstitutional practitioner for health management or disease treatment [11]. The questionnaire is presented in Supplementary Appendix 1.

This study was approved by the Ethics Committee of the Wonkwang University Hospital (WKIRB 2013-3). Data were collected in 2013.

### 2.3. Statistical Analysis

Microsoft Excel software (Microsoft Office Professional Plus 2013, United States) was used for data entry, data documentation, and descriptive statistical analysis [12]. Individual CAM use was categorised into 3 types following the National Centre for Complementary and Integrative Health (NCCIH); natural products, mind and body medicine, and other complementary health approaches. CAM use among respondents was measured as a percentage.

## 3. Results

A total of 55 participants were included in the survey (men: 39 (70.9%); women: 16 (29.1%); limb type: 47 (85.5%); bulbar type: 6 (10.9%); mean age: 55.96 ± 11.31 years; duration from onset: 3.74 ± 4.04 years; duration from diagnosis: 2.72 ± 3.56 years). The most common onset symptom was limb weakness (42.9%), followed by fasciculation (14.3%) and muscle cramps (10.2%). Only 1 (1.8%) participant was married, and 27 (49.1%) were religious; urban dwellers constituted the majority (*n* = 23, 41.8%) of the population.

Forty-three (78.1%) of the participants were high school or college graduates, and the monthly household income was mostly between 1,010,000 and 2,000,000 Korean won (KRW; *n* = 18, 32.7%). In terms of past or present occupation, office workers constituted the majority (*n* = 33, 60.0%) of the population (Table 1).

### 3.1. Prevalence and Pattern of CAM Use

Among the 55 survey respondents, all had used more than 1 CAM therapy for the symptoms related to ALS. The average number of CAM treatment methods used was 3.54 per person.

A total of 195 CAM types were used by the respondents. Mind and body medicine (50.3%) was the most commonly used category followed by natural products (46.7%) (Table 2). Among natural products, dietary treatments (23.6%) were the most commonly used. Among mind and body medicine, massage (9.7%) was the most commonly used (Supplementary Appendix 2).

The main reason for initiating CAM use was the vague expectation that symptoms would improve (*n* = 40, 72.7%). This was followed by poor results from conventional medicine (*n* = 9, 16.4%) and the high expense of conventional medicine (*n* = 6, 10.9%). Users expected increase in physical strength in most cases (*n* = 24, 43.6%), followed by reduction in disease progression (*n* = 23, 41.8%) and strengthening of muscles (*n* = 18, 32.7%). Among 195 cases of CAM use, patients were unsure about the effects in 146 cases (74.9%); most of the others stated that they had experienced the effects (*n* = 39, 20%). Thirty-four (61.8%) patients had previously discontinued CAM use. Among them, 70.6% had discontinued CAM because of unsatisfactory effects. Most participants were not sure about recommending CAM to other patients (*n* = 24, 43.6%) (Table 2).

### 3.2. Duration of and Expenditure for CAM Use

In the 195 cases of CAM use, the mean expenditure was 288,385.28 ± 685,265.14 won ($249.58) per month and the mean duration of CAM use was 11.54 ± 20.09 months.

### 3.3. Origins of CAM Use

Of the 55 respondents, 35 (63.6%) obtained information from family members or friends. This was followed by the Internet, mass media, and books (*n* = 11, 20.0%) and healthcare providers such as doctors (*n* = 4, 7.3%).

When using CAM, 15 patients (25%) did not consult anyone, 15 (25%) consulted family members, and 12 (22%) consulted authorised healthcare providers. The most commonly cited reason for not consulting with healthcare providers was “no need to consult with healthcare providers” (*n* = 22, 51.2%), followed by “not asked about CAM use by healthcare providers” (*n* = 7, 16.3%) and “fear of CAM use restriction by healthcare providers” (*n* = 5, 11.6%) (Table 3).

### 3.4. Adverse Effects of CAM among Patients with ALS

Among 55 patients, 11 (20%) experienced adverse effects from CAM use for a total of 22 events. Most adverse effects were mild (*n* = 16, 72.7%), and most patients continued using the same CAM method (*n* = 14, 63.6%). Most patients responded that they were recovering from the adverse effects (*n* = 10, 45.5%) (Table 4).

## 4. Discussion

This study, involving a survey regarding CAM use in ALS patients, is the only nationwide survey available in South Korea. It provides preliminary evidence for the extent, types, motives, expenditure, and adverse effects of CAM use.

The type or prevalence of CAM tends to depend on the country [7]. Similar to a survey in Shanghai, China, all participants used CAM for symptoms related to ALS. We speculate that such a high prevalence is partly due to the Korean healthcare system. Provisions for traditional forms of medicine and medical treatment, such as acupuncture and herbal therapy, are an accepted part of conventional health care and are covered by the national healthcare system in Korea [13]. Therefore, South Koreans are familiar with CAM. Dietary treatment or functional foods are popular forms of CAM. Perhaps, CAM users consider these therapies only as food and perceive that there is no associated risk.

Here, we defined CAM as the recommendation or administration of a procedure by a noninstitutional practitioner for health management. Acupuncture, cupping, or Chuna manipulative treatment can be covered by an authorized Korean medicine doctor. The rate of these types of treatments is relatively low compared to that in the survey in Germany [7].

The main reason for CAM use was a vague expectation of improvement, though patients were unsure about its effectiveness; nevertheless, they used CAM for an average of 11.54 months. Perhaps, the concept of “hope” is fundamental among the reasons for seeking CAM [14].

Regarding consultation, 25% of the patients did not consult with anyone about CAM, with many of those patients stating that there was “no need to consult with healthcare providers” (51.2%). These results coincide with a previous study indicating that ALS patients have significant levels of nondisclosure to clinicians regarding their CAM use [3]. This may reflect a desire to avoid conflict or embarrassment; also, the response and attitude of the healthcare providers towards CAM may play a significant role in ALS patient decision-making concerning CAM use [6]. Among patients who experienced adverse effects from CAM use, most patients continued using the same type of CAM (63.6%). Interestingly, most patients responded that they were in a state of recovery from the adverse effects (45.5%). This finding indicates that physical and psychological harm may come to patients with ALS via CAM use [4]. Shared decision-making is preferred by both patients and doctors and is associated with improved compliance and better health outcomes in doctor-patient relationships [4]. Therefore, healthcare providers should ask about and discuss CAM use and its adverse effects in a supportive and open manner [6].

The effects of most CAM therapies remain to be elucidated. The ALS research group has built an interactive program known as “ALS Untangled” to identify any potential benefits or harm of CAM [15]. The ALS Untangled team has published several narrative reviews on the effects and risks of various CAM methods for ALS, and there is only 1 review paper focusing upon the clinical efficacy of CAM for ALS [4]. This review reported the effects of CAM as generally unproven and highlighted conflicting evidence regarding the effect of dietary supplements and other options for ALS management. Further evidence for the efficacy of CAM is required considering the high prevalence of CAM use among ALS patients.

This study has several limitations. The sample size is small with respect to the prevalence of ALS, and the sample only includes patients in late stages of ALS who use a ventilator; nonetheless, this study is significant because it is the first survey conducted in South Korea regarding CAM use among ALS patients.

## 5. Conclusion

In conclusion, CAM use is highly prevalent among ALS patients in South Korea. Healthcare providers should inquire about CAM use and give accurate information about it in an open manner. Further evidence for the efficacy of CAM is required, and specific guidance for consulting with ALS patients concerning CAM use should be developed in future studies.

## Figures and Tables

**Table 1:** Characteristics of participants (*n* = 55).

Characteristics	*n* (%)
Age (years)	55.96 ± 11.31
30–39	5 (9.1)
40–49	9 (16.4)
50–59	18 (32.7)
60–69	17 (30.9)
70–79	5 (9.1)
80–89	1 (1.8)
Sex	
Male	39 (70.9)
Female	16 (29.1)
Type	
Limb type	47 (85.5)
Bulbar type	6 (10.9)
Missing data	2 (3.6)
Duration from onset (years)	3.74 ± 4.04
Duration from diagnosis (years)	2.72 ± 3.56
Onset symptom^*∗*^ (*n* = 98)	
Limb weakness	42 (42.9)
Fasciculation	14 (14.3)
Muscle cramps	10 (10.2)
Numbness	9 (9.2)
Weight loss	9 (9.2)
Dysarthria	7 (7.1)
Pain	5 (5.1)
Loss of appetite	1 (1.0)
Difficulty swallowing	1 (1.0)
Marital status	
Married	1 (1.8)
Not married	50 (90.9)
Others (divorced, etc.)	4 (7.3)
Religious	
Yes	27 (49.1)
No	24 (43.6)
Missing data	4 (7.3)
Region	
Metropolitan	20 (36.4)
Town	23 (41.8)
Village	10 (18.2)
Missing data	2 (3.6)
Level of education	
No high school diploma	12 (21.8)
High school diploma	22 (40.0)
College or university	21 (38.2)
Occupation (past or present)	
Office worker	33 (60.0)
Manual labour	15 (27.3)
Others	6 (10.9)
Missing data	1 (1.8)
Monthly household income^†^	
≤200	30 (54.5)
201–300	8 (14.5)
301–400	7 (12.7)
≥400	10 (18.2)

^*∗*^Allowed duplicate responses; ^†^unit: 10,000 KRW.

**Table 2:** Prevalence and pattern of CAM use (*n* = 55).

Questionnaire	*n* (%)
CAM type (*n* = 195)	
Natural products	91 (46.7)
Mind and body medicine	98 (50.3)
Others	6 (3.1)
Main reason for CAM use (*n* = 55)	
Vague expectations from CAM	40 (72.7)
Poor results from conventional medicine	9 (16.4)
High expense of conventional medicine	6 (10.9)
Expectations from CAM use^*∗*^ (*n* = 55)	
Increasing physical strength	24 (43.6)
Arrest of disease progression	23 (41.8)
Strengthening of muscles	18 (32.7)
Psychological stability	13 (23.6)
Promoting immune function	11 (20)
Pain	7 (12.7)
Insomnia	4 (7.3)
Others	5 (9.1)
Subjective effects (*n* = 195)	
Being effective	39 (20.0)
Unsure about effectiveness	146 (74.9)
Disease progression	2 (1.0)
Adverse effects	8 (4.1)
Experience of discontinuing CAM use (*n* = 55)	
Yes	34 (61.8)
No	19 (34.5)
Missing data	2 (3.6)
Reasons for discontinuing CAM use (*n* = 34)	
No satisfactory effect with CAM	24 (70.6)
Restrained by healthcare providers	3 (8.8)
Adverse effects with CAM	3 (8.8)
Inconvenience of CAM use (time, distance, etc.)	3 (8.8)
Doubt of CAM effectiveness	1 (2.9)
Intention of recommending CAM use (*n* = 55)	
Yes	8 (14.5)
No	23 (41.8)
Do not know	24 (43.6)

^*∗*^Allowed duplicate responses; CAM, complementary alternative medicine; ALS, amyotrophic lateral sclerosis.

**Table 3:** Origins of CAM use (*n* = 55).

Questionnaire	*n* (%)
Sources of information on CAM (*n* = 55)	
Family members or friends	35 (63.6)
Internet, mass media, and books	11 (20.0)
Healthcare providers	4 (7.3)
Noninstitutional CAM practitioners	1 (1.8)
Others	1 (1.8)
Missing data	3 (5.5)
Counselling for CAM use (*n* = 55)	
None	15 (27.3)
Family members	15 (27.3)
Healthcare providers	12 (21.8)
Unauthorized CAM practitioners	7 (12.7)
Patients with ALS	4 (7.3)
Others	2 (3.6)
Reason for not choosing healthcare providers as counsellors (*n* = 43)	
No need to consult with healthcare providers	22 (51.2)
Healthcare providers have not inquired about CAM use	7 (16.3)
Fear of restriction of CAM use by healthcare providers	5 (11.6)
No time for consultation	3 (7.0)
Others	5 (11.6)
Missing data	1 (2.3)

**Table 4:** Adverse effects of CAM use among patients with ALS (*n* = 22).

Questionnaire	*n* (%)
Type of adverse effect	
Systemic reaction	2 (9.1)
Skin and appendix	1 (4.5)
Eye/nose/ear/mouth	1 (4.5)
Cardiovascular system	1 (4.5)
Gastrointestinal tract	3 (13.6)
Liver and biliary tract	1 (4.5)
Respiratory system	2 (9.1)
Mental and behavioural disorders	2 (9.1)
Endocrine and genitourinary system	1 (4.5)
Musculoskeletal disorders	4 (18.2)
Others	4 (18.2)
Severity of adverse effects	
Mild^*∗*^	16 (72.7)
Moderate^†^	4 (18.2)
Severe^§^	2 (9.1)
Changes of CAM use after adverse effects	
Maintaining dosage and procedure	14 (63.6)
Reducing dosage and number of procedures	2 (9.1)
Stopping CAM use	6 (27.3)
Recovery of adverse effects	
Recovered	8 (36.4)
In the state of recovery	10 (45.5)
Not recovered (aftereffects remain)	3 (13.6)
Missing data	1 (4.5)

CAM, complementary and alternative medicine; ALS, amyotrophic lateral sclerosis. ^*∗*^Cases wherein adverse effects do not significantly interfere with normal life; patients do not require treatment. ^†^Cases with significantly affected normal life; patients may require treatment or may have recovered after treatment. ^§^Cases requiring a high degree of treatment; the aftereffects remain.

## Data Availability

The data used to support the findings of this study are available from the corresponding author upon request.

## Supplementary Materials

Supplementary Materials Supplementary Appendix 1: a questionnaire survey conducted to understand the treatment behaviours relating to ALS and muscle dystrophy in ALS patients. Based on the results of this survey, we aimed to ascertain the precise state of methods used by ALS patients in Korea to treat the disease and improve health, hoping that data will be used to establish policies that improve treatments for ALS patients in Korea. Supplementary Appendix 2: prevalence of CAM use by CAM type (*n* = 195). Supplementary Appendix 3: used modalities in dietary treatments (*n* = 46).

## References

[1] Land M. H., Wang J. (2018). Complementary and alternative medicine use among allergy practices: results of a nationwide survey of allergists. *Journal of Allergy and Clinical Immunology: In Practice*.

[2] World Health Organization (2013). *WHO Traditional Medicine Strategy 2014–2023*.

[3] Adams J., Lee M., Peng W. (2018). Critical review of complementary and alternative medicine use in amyotrophic lateral sclerosis: prevalence and users’ profile, decision-making, information seeking, and disclosure in the face of a lack of efficacy. *Neurodegenerative Diseases*.

[4] Bedlack R. S., Joyce N., Carter G. T., Paganoni S., Karam C. (2015). Complementary and alternative therapies in amyotrophic lateral sclerosis. *Neurologic Clinics*.

[5] Astin J. A. (1998). Why patients use alternative medicine: results of a national study. *JAMA*.

[6] Kim S., Chung S. E., Lee S., Park J., Choi S., Kim S. (2016). Experience of complementary and alternative medicine in patients with amyotrophic lateral sclerosis and their families: a qualitative study. *Amyotrophic Lateral Sclerosis and Frontotemporal Degeneration*.

[7] Wasner M., Klier H., Borasio G. D. (2001). The use of alternative medicine by patients with amyotrophic lateral sclerosis. *Journal of the Neurological Sciences*.

[8] Vardeny O., Bromberg M. B. (2005). The use of herbal supplements and alternative therapies by patients with amyotrophic lateral sclerosis (ALS). *Journal Of Herbal Pharmacotherapy*.

[9] Pan W., Chen X., Bao J. (2013). The use of integrative therapies in patients with amyotrophic lateral sclerosis in Shanghai, China. *Evidence-Based Complementary and Alternative Medicine*.

[10] Moon S., Baek S., Park J. (2012). The use of complementary and alternative medicine in patients with type 2 diabetes mellitus: community based survey. *Journal of Internal Korean Medicine*.

[11] Baek S.-M., Choi S. M., Seo H.-J. (2013). Use of complementary and alternative medicine by self-or non-institutional therapists in South Korea: a community-based survey. *Integrative Medicine Research*.

[12] Levine D. M. (2016). *Statistics for Managers Using Microsoft Excel: Student Value Edition*.

[13] Kim S., Kim B., Mun S. (2016). Development of a template for the classification of traditional medical knowledge in Korea. *Journal of Ethnopharmacology*.

[14] Puataweepong P., Sutheechet N., Ratanamongkol P. (2012). A survey of complementary and alternative medicine use in cancer patients treated with radiotherapy in Thailand. *Evidence-Based Complementary and Alternative Medicine*.

[15] Bedlack R., Hardiman O. (2009). ALSUntangled (ALSU): A scientific approach to off-label treatment options for people with ALS using tweets and twitters. *Amyotrophic Lateral Sclerosis*.

