# Supplementary material for: Complementary and Alternative Medicine Use in Amyotrophic Lateral Sclerosis Cases in South Korea
Source: Evid Based Complement Alternat Med. 2019 Jul 25;2019:4217057. doi: 10.1155/2019/4217057 (PMC6683772; doi:10.1155/2019/4217057)
Supplement: Supplementary Materials — Supplementary Appendix 1: a questionnaire survey conducted to understand the treatment behaviours relating to ALS and muscle dystrophy in ALS patients. Based on the results of this survey, we aimed to ascertain the precise state of methods used by ALS patients in Korea to treat the disease and improve health, hoping that data will be used to establish policies that improve treatments for ALS patients in Korea. Supplementary Appendix 2: prevalence of CAM use by CAM type (n = 195). Supplementary Appendix 3: used modalities in dietary treatments (n = 46). [file 4217057.f1.zip › 4217057.f1/Supplemental Appendix 1.docx]

|  | Time taken: _______mins | \| ID \|  \|  \| - \|  \|  \|  \| \| --- \| --- \| --- \| --- \| --- \| --- \| --- \| |
| --- | --- | --- | --- | --- | --- | --- | --- | --- | --- |

| **Questionnaire to Examine Usage of Folk Therapy by Patients with Amyotrophic Lateral Sclerosis (ALS)** |
| --- |

| Hello,  We are conducting a questionnaire survey to understand treatment behaviours relating to ALS and muscle dystrophy in ALS patients.  Based on the results of this survey, we aim to ascertain the precise state of methods used by ALS patients in Korea to treat the disease and improve health, and we hope that this data will be used to establish policies that improve treatments for ALS patients in Korea.  Since privacy is guaranteed for your responses based on Articles 33 and 34 of the Statistics Act, and your responses will only be used for the purposes of this survey, please respond sincerely. Thank you very much. | | | |
| --- | --- | --- | --- |
| **▢ Study period: Sep – Nov, 2013**  **▢ Study center: Gwangju Medical Center, Wonkwang University** | 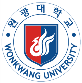 | **▢ Principal investigator: Sungchul Kim**  **▢ Subinvestigator: Sungha Kim**  (Contact No.: 042-868-9385) | 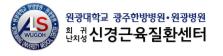 |

**** Please fill in the respondent’s details.**

| **Respondent Name** |  | **Telephone No.** |  |
| --- | --- | --- | --- |
| **Respondent Age** | ___ years | **Respondent Gender** | 1) Male 2) Female |
| **Area of Residence** | 1) Seoul 2) Busan 3) Daegu 4) Incheon 5) Gwangju 6) Daejeon 7) Ulsan 8) Gyeonggi-do 9) Gangwon-do 10) Chungcheongbuk-do 11) Chungcheongnam-do  12) Jeollabuk-do 13) Jeollanam-do 14) Gyeongsanbuk-do 15) Gyeongsannam-do 16) Jeju-do | | |
| **Unit of Area of Residence** | 1) Metropolitan city 2) Small-to-medium city 3) *Eup*/*myeon* | | |

| Respondent’s demographic characteristics |
| --- |

DQ1. What is your marital status?

1) Unmarried 2) Currently married 3) Other (bereaved, divorced, etc.)

DQ2. What was the highest level of education that you completed? (Dropping out is not counted as graduation)

1) No formal education or elementary school dropout 2) Elementary school graduation 3) Middle school graduation

4) High school graduation 5) College graduation 6) Graduate school graduation or higher

DQ3. What was your previous occupation?

1) Management or administration 2) Professional occupation 3) Office worker 4) Service worker

5) Salesperson 6) Agriculture or fisheries 7) Technical occupation 8) Simple labour

9) Military occupation 10) Student 11) Homemaker 12) Other (Please specify: _________)

DQ4. Do you consider yourself religious?

1) Yes 2) No 3) No comment

DQ5. What is your average monthly household income?

1) ≤1 million KRW 2) 1.01 – 2 million KRW 3) 2.01 – 3 million KRW

4) 3.01 – 4 million KRW 5) ≥4.01 million KRW

DQ6. Do any of your family work in healthcare and medicine? (**You may select multiple answers**)

1) Doctor 2) Korean Medicine doctor 3) Nurse 4) Pharmacist 5) None

| Respondent’s medical history |
| --- |

HQ1. Have you been diagnosed with ALS (motor neurone disease) by a doctor?

1) Yes ☞ HQ1-1 2) No → **End of questionnaire**

HQ2. In which area did you first experience symptoms?

1) Upper limbs 2) Lower limbs 3) Language disorder 4) Other ___________

HQ3. What were your symptoms **at the time of onset**? (**You may select multiple answers**)

1) Weakness of the arms and legs 2) Dysarthria (stuttering) 3) Muscle tremor (spasm)

4) Tingling in the arms and legs 5) Pain 6) Muscle cramps

7) Reduced appetite 8) Weight loss 9) Breathing difficulties

10) Other ___________

HQ4. When did you first develop symptoms? ______ years ago (Ex: I first developed symptoms 5 **years ago**)

HQ5. When were you diagnosed with ALS? ______ years ago (Ex: I was diagnosed 3 **years ago**)

HQ6. What is the state of your current ALS treatment?

1) I am only receiving outpatient tests 2) I regularly receive inpatient treatment or outpatient treatment at the hospital

3) I visit the hospital for treatment and management 4) I am in a convalescent hospital

5) I have given up hospital treatment and am at home 6) Not sure

| Usage of various therapies outside of western and Korean Medicine hospitals and clinics |
| --- |

| The purpose of this questionnaire is to investigate only **therapies recommended/performed by non-institutionalized practitioners** or **self-treatment** methods for health management or disease treatment **outside of western and Korean Medicine hospitals and clinics**.  **★★★** We are only investigating therapies used for **ALS or the treatment of symptoms caused by ALS**.  Please be careful **not to include overly common methods (everyday methods or foods/medicines)**. |
| --- |

[For Q1-1. – Q1-10., you should **refer to the table** for your responses. **The table shows the 32 categories of therapy**.

**Please look at the table on the next page** and make your responses.

Q1-1. Which of the following therapies have you used to try to treat **ALS** or to alleviate **symptoms** caused by ALS?

Q1-2. With reference to the types of therapy, please write the name or number of the main therapy that you have actually used.

* Please only include therapies (treatment modalities, food, or medications) used to alleviate ALS, muscle dystrophy, or resulting symptoms). Please exclude everyday therapies (treatment modalities, food, or medications)

Q1-3. Please describe in detail the “what”, “how” and “why” of the therapy.

**(Ex. What: Chaga mushroom/ How: Boiling to make tea and drinking several times per day/ Why: To improve my vitality)**

Q1-4. When did you first use this therapy?

Q1-5. When you used this therapy, did you perform it yourself, or was it performed by a practitioner? (If it was performed by family or a friend, this is included in self-administration)

Q1-6. Do you consider this a traditional therapy that was started and developed in Korea?

Q1-7. When you use this therapy, do you think, subjectively, that it is effective?

Q1-8. In total, for how long have you used this therapy?

Q1-9. How much has it cost you, **per month**, to use this therapy?

Q1-10. [For therapies 16–27 only] Did you only take one type of medication or food, or did you use several medications or foods in combination?

| Type of therapy | Q1-1.  I used this therapy. | Q1-2.  What was the name of the main therapy you used?  (Please enter the number with reference to the ‘Type of therapy’ column) | Q1-3.  Please describe the method used for the therapy.  (What, How, and Why) | Q1-4. When did you first use the therapy?  1) Before diagnosis  2) From diagnosis to 6 months after diagnosis  3) 6 months to 1 year after diagnosis  4) More than 1 year after diagnosis | Q1-5.  Who administered the therapy?  1) Self-administered  (including family)  2) Practitioner  3) Both | Q1-6.  Was it a traditional therapy?  1) Traditional  2) Of foreign origin  3) Not sure | Q1-7  Please indicate whether you thought the therapy was effective, or whether there were adverse effects.  1) The therapy was effective  2) Not sure (no effect)  3) My condition worsened  4) I experienced adverse effects | Q1-8  How long, in total, did you use the therapy?  (months) | Q1-9  How much did the therapy cost?  (KRW / month) | Q1-10  Did you take the medication alone or in combination with other medications?  (#16–27)  ① Single medication  ② Combination |
| --- | --- | --- | --- | --- | --- | --- | --- | --- | --- | --- |
| 1. **Acupuncture** (**outside** of a Korean Medicine hospital or clinic) | □ |  |  | 1) 2) 3) 4) | 1) 2) 3) | 1) 2) 3) | 1) 2) 3) 4) | ______ months | ______ KRW  / 1 month |  |
| Ex: 1) Standard acupuncture  2) Hand acupuncture  3) Gold thread acupuncture  4) Thread-embedding acupuncture  5) Other |  |  |  |  |  |  |  |  |  |  |
| 2. **Moxibustion** (**outside** of a Korean Medicine hospital or clinic) | □ |  |  | 1) 2) 3) 4) | 1) 2) 3) | 1) 2) 3) | 1) 2) 3) 4) | ______ months | ______ KRW  / 1 month |  |
| 3. **Cupping** (**outside** of a Korean Medicine hospital or clinic) | □ |  |  | 1) 2) 3) 4) | 1) 2) 3) | 1) 2) 3) | 1) 2) 3) 4) | ______ months | ______ KRW  / 1 month |  |
| 4. **Bloodletting** (**outside** of a Korean Medicine hospital or clinic) | □ |  |  | 1) 2) 3) 4) | 1) 2) 3) | 1) 2) 3) | 1) 2) 3) 4) | ______ months | ______ KRW  / 1 month |  |
| 1) Pricking the finger for indigestion  2) *Simcheon* bloodletting 3) Other |  |  |  |  |  |  |  |  |  |  |
| 5. ***Chuna* therapy** (**outside** of a Korean Medicine hospital or clinic) | □ |  |  | 1) 2) 3) 4) | 1) 2) 3) | 1) 2) 3) | 1) 2) 3) 4) | ______ months | ______ KRW  / 1 month |  |
| 6. **Chiropractic, osteopathy, and taping** (**outside** of a Korean Medicine hospital or clinic) | □ |  |  | 1) 2) 3) 4) | 1) 2) 3) | 1) 2) 3) | 1) 2) 3) 4) | ______ months | ______ KRW  / 1 month |  |
| 1) Chiropractic  2) Spinal correction  3) Disc correction  4) Osteopathy  5) Osteopathic manipulation  6) Kinesio-taping  7) Other |  |  |  |  |  |  |  |  |  |  |
| 7. **Acupressure, foot massage, tapping therapy** | □ |  |  | 1) 2) 3) 4) | 1) 2) 3) | 1) 2) 3) | 1) 2) 3) 4) | ______ months | ______ KRW  / 1 month |  |
| 1) Meridian massage  2) Massage  3) He Gu (LI4) pressure  4) Foot massage  5) Foot reflexology  6) Meridian tapping  7) Other |  |  |  |  |  |  |  |  |  |  |
| 8. **Thermotherapy** | □ |  |  | 1) 2) 3) 4) | 1) 2) 3) | 1) 2) 3) | 1) 2) 3) 4) | ______ months | ______ KRW  / 1 month |  |
| 1) Lower-body bathing  2) Abdominal bathing  3) Steam therapy  4) Hot packs  5) Hot spring bathing  6) Sauna  7) Red clay sauna  8) Other |  |  |  |  |  |  |  |  |  |  |
| 9. **Exercise therapy** | □ |  |  | 1) 2) 3) 4) | 1) 2) 3) | 1) 2) 3) | 1) 2) 3) 4) | ______ months | ______ KRW  / 1 month |  |
| 1) Pilates  2) Goldfish exercise  3) Exercise of joined palms and joined soles  4) 108 bows  5) Other |  |  |  |  |  |  |  |  |  |  |
| 10. **Physical therapy** using home exercise devices | □ |  |  | 1) 2) 3) 4) | 1) 2) 3) | 1) 2) 3) | 1) 2) 3) 4) | ______ months | ______ KRW  / 1 month |  |
| 1) Low-frequency therapeutic device  2) Magnetic jade mat  3) Other |  |  |  |  |  |  |  |  |  |  |
| 11. **Qi therapy**  *※Receiving qi from a qi therapist* | □ |  |  | 1) 2) 3) 4) | 1) 2) 3) | 1) 2) 3) | 1) 2) 3) 4) | ______ months | ______ KRW  / 1 month |  |
| 12. **Breath training, meditation, yoga**  *※ Self-directed* | □ |  |  | 1) 2) 3) 4) | 1) 2) 3) | 1) 2) 3) | 1) 2) 3) 4) | ______ months | ______ KRW  / 1 month |  |
| 1) Deep breathing  2) Tanhak meditation centre  3) Seokmun breathing  4) Zen meditation  5) Yoga  6) Other |  |  |  |  |  |  |  |  |  |  |
| 13. **Spiritual therapy** | □ |  |  | 1) 2) 3) 4) | 1) 2) 3) | 1) 2) 3) | 1) 2) 3) 4) | ______ months | ______ KRW  / 1 month |  |
| 1) Exorcism  2) *Gut*  3) Hypnosis  4) Consultation with a shaman  5) Laying on of hands  6) Other |  |  |  |  |  |  |  |  |  |  |
| 14. **Activity therapy**  *※ Not recommended or prescribed by a doctor or Korean Medicine doctor* | □ |  |  | 1) 2) 3) 4) | 1) 2) 3) | 1) 2) 3) | 1) 2) 3) 4) | ______ months | ______ KRW  / 1 month |  |
| 1) Music therapy  2) Dance therapy  3) Horticultural therapy  4) Other |  |  |  |  |  |  |  |  |  |  |
| 15. **Forest therapy** | □ |  |  | 1) 2) 3) 4) | 1) 2) 3) | 1) 2) 3) | 1) 2) 3) 4) | ______ months | ______ KRW  / 1 month |  |
| 1) Forest immersion  2) Wind bathing  3) Other |  |  |  |  |  |  |  |  |  |  |
| 16. **Green vegetable juice dietary therapy** | □ |  |  | 1) 2) 3) 4) | 1) 2) 3) | 1) 2) 3) | 1) 2) 3) 4) | ______ months | ______ KRW  / 1 month | ① ② |
| 1) Kale  2) Ashitaba (*myeongil-cho*)  3) Ashitaba (*Sinseon-cho*)  4) Packaged green vegetable juice  5) Other |  |  |  |  |  |  |  |  |  |  |
| 17. **Fruit juice dietary therapy** | □ |  |  | 1) 2) 3) 4) | 1) 2) 3) | 1) 2) 3) | 1) 2) 3) 4) | ______ months | ______ KRW  / 1 month | ① ② |
| 1) Grape juice  2) Pear juice  3) Korean blackberry juice  4) Pumpkin juice  5) Other |  |  |  |  |  |  |  |  |  |  |
| 18. **Mushroom-based dietary therapy**  *※Excluding mushrooms that have been released in the form of products* | □ |  |  | 1) 2) 3) 4) | 1) 2) 3) | 1) 2) 3) | 1) 2) 3) 4) | ______ months | ______ KRW  / 1 month | ① ② |
| 1) *Sanghwang* mushrooms (*Phellinus linteus*)  2) Agaricus mushrooms  3) Shiitake mushrooms  4) Lingzhi mushrooms  5) Chaga mushrooms  6) Other |  |  |  |  |  |  |  |  |  |  |
| 19. **Wild herb and seaweed-based dietary therapy**  *※ Excluding commonly eaten side dishes* | □ |  |  | 1) 2) 3) 4) | 1) 2) 3) | 1) 2) 3) | 1) 2) 3) 4) | ______ months | ______ KRW  / 1 month | ① ② |
| 20. **Charcoal or bamboo salt-based dietary therapy** | □ |  |  | 1) 2) 3) 4) | 1) 2) 3) | 1) 2) 3) | 1) 2) 3) 4) | ______ months | ______ KRW  / 1 month | ① ② |
| 1) Charcoal  2) Bamboo salt  3) Other |  |  |  |  |  |  |  |  |  |  |
| 21. **Animal-based dietary therapy**  *※ Excluding foods traditionally eaten on special days (e.g., samgyetang on bok-nal)* | □ |  |  | 1) 2) 3) 4) | 1) 2) 3) | 1) 2) 3) | 1) 2) 3) 4) | ______ months | ______ KRW  / 1 month | ① ② |
| 1) Dog elixir  2) Carp juice  3) Black goat  4) Silkworm pupae  5) Common octopus  6) Chicken feet  7) Duck  8) Health-boosting foods  9) Other |  |  |  |  |  |  |  |  |  |  |
| 22. **Colonic irrigation** | □ |  |  | 1) 2) 3) 4) | 1) 2) 3) | 1) 2) 3) | 1) 2) 3) 4) | ______ months | ______ KRW  / 1 month | ① ② |
| 1) Coffee irrigation  2) Other |  |  |  |  |  |  |  |  |  |  |
| 23. **Specific dietary therapies**  *※ Excluding low-sodium diets, high-protein diets, etc. advised by a doctor* | □ |  |  | 1) 2) 3) 4) | 1) 2) 3) | 1) 2) 3) | 1) 2) 3) 4) | ______ months | ______ KRW  / 1 month | ① ② |
| 1) Constitution-based diet  2) Raw diet  3) Zen diet  4) One food diet  5) Fasting  6) Five elements diet  7) Other |  |  |  |  |  |  |  |  |  |  |
| 24. **Medicinal tea**  *※ Medicinal herbs in the form of tea* | □ |  |  | 1) 2) 3) 4) | 1) 2) 3) | 1) 2) 3) | 1) 2) 3) 4) | ______ months | ______ KRW  / 1 month | ① ② |
| 1) Green tea  2) Solomon’s seal  3) Other |  |  |  |  |  |  |  |  |  |  |
| 25. **Medicinal spirits**  *※ Medicinal herbs in the form of alcohol* | □ |  |  | 1) 2) 3) 4) | 1) 2) 3) | 1) 2) 3) | 1) 2) 3) 4) | ______ months | ______ KRW  / 1 month | ① ② |
| 26. **Herbal medicines** (Outside of a Korean Medicine hospital or clinic)  *※ Only if bought and prepared directly* | □ |  |  | 1) 2) 3) 4) | 1) 2) 3) | 1) 2) 3) | 1) 2) 3) 4) | ______ months | ______ KRW  / 1 month | ① ② |
| 27. **Health-boosting foods based on medicinal herbs** | □ |  |  | 1) 2) 3) 4) | 1) 2) 3) | 1) 2) 3) | 1) 2) 3) 4) | ______ months | ______ KRW  / 1 month | ① ② |
| 1) Red ginseng  2) Cordyceps  3) Mushroom products  4) Other |  |  |  |  |  |  |  |  |  |  |
| 28. **Health supplements**  *※ Medicinal herbs in the form of packaged pills* | □ |  |  | 1) 2) 3) 4) | 1) 2) 3) | 1) 2) 3) | 1) 2) 3) 4) | ______ months | ______ KRW  / 1 month |  |
| 1) Vitamins  2) Saw palmetto  3) Omega-3  4) Other |  |  |  |  |  |  |  |  |  |  |
| 29. **Natural external applications** | □ |  |  | 1) 2) 3) 4) | 1) 2) 3) | 1) 2) 3) | 1) 2) 3) 4) | ______ months | ______ KRW  / 1 month |  |
| 1) Liquid smoke  2) Chameleon plant  3) Persimmon leaves  4) Other |  |  |  |  |  |  |  |  |  |  |
| 30. **Artificial external applications** | □ |  |  | 1) 2) 3) 4) | 1) 2) 3) | 1) 2) 3) | 1) 2) 3) 4) | ______ months | ______ KRW  / 1 month |  |
| 1) Softened water  2) Chlorinated water  3) Bath soaks  4) Other |  |  |  |  |  |  |  |  |  |  |
| 31. **Aromatherapy** | □ |  |  | 1) 2) 3) 4) | 1) 2) 3) | 1) 2) 3) | 1) 2) 3) 4) | ______ months | ______ KRW  / 1 month |  |
| 1) Aromatherapy  2) Other |  |  |  |  |  |  |  |  |  |  |
| 32. **Other therapies** | □ |  |  | 1) 2) 3) 4) | 1) 2) 3) | 1) 2) 3) | 1) 2) 3) 4) | ______ months | ______ KRW  / 1 month |  |
| Other therapies that were used, but were not included in categories 1 to 31 above  Ex: New Start Therapy, Nishi Shiki, etc. |  |  |  |  |  |  |  |  |  |  |

Q2. Have you ever **used even** one of the 32 types of therapy in the table above?

1) Yes, I have used one or more. **☞ Q3.**  2) No, I haven’t even used one. **☞ Q2-1.**

Q2-1. What is the main reason that you have not used any of the methods listed above?

1) A doctor/Korean Medicine doctor forbade use of these therapies 2) I have never heard of the therapy

3) I can’t trust the effectiveness of the therapy 4) I’m worried about adverse effects

5) Treatment at the western or Korean Medicine hospital or clinic was sufficient 6) Because of the financial burden

7) Other (Please specify: ___________)  **==> (Non-users) End of questionnaire**

Q3. Have you ever **started and then stopped** one of the above therapies? 1) Yes **☞ Q3-1** 2) No

Q3-1. What was **the main** reason that you stopped using the therapy?

1) A doctor/Korean Medicine doctor forbade its use 2) The therapy did not show a satisfactory effect

3) The therapy caused adverse effects 4) The therapy was not convenient (e.g., in terms of time or travel distance)

5) Because of the financial burden 6) Because I achieved the purposes of the treatment

7) Other (Please specify: ________________)

| Reasons for usage and routes of access for various therapies outside of western and Korean Medicine hospitals and clinics |
| --- |

Q4. What was your main reason for selecting the above therapies to treat ALS?

1) Conventional hospital treatment caused severe adverse effects 2) Conventional hospital treatment was not helping

3) A vague sense of expectation about treatment outside of a conventional hospital 4) Conventional hospital treatment was too expensive

5) Other (Please specify: ______________________)

Q5. What effects were you expecting when you used the above therapies to treat ALS outside of western and Korean Medicine hospitals and clinics? (You may select multiple answers)

1) Muscle strengthening 2) Improved vitality 3) Improved immune function 4) Pain relief

5) Insomnia relief 6) Slowing disease progression 7) Psychological relief 8) Other (Please specify: ____________________)

Q6. What was **the main** route by which you learned about the above therapies?

1) Family and relatives 2) Friends and acquaintances

3) The Internet 4) Health-related reading materials

5) Recommended by a western or Korean Medicine doctor 6) Recommended by a pharmacist

7) Recommended by a practitioner related to the therapy 8) Public media (TV, radio, etc.) 9) Other (Please specify:___________)

Q7. Who did you consult with then use decided to use the above therapies?

1) Western or Korean Medicine doctor 2) Pharmacist 3) Non-institutionalized therapist (practitioner)

4) Family 5) Patient with the same disease 6) Other (Please specify: ___________)

7) Nobody

Q7-1. If you did not choose a western or Korean Medicine doctor as your main consultant (i.e., **if you did not answer (1) for Q7**), what was **the main** reason for this?

1) The doctor did not ask whether I was using any therapies

2) I didn’t feel that I needed to consult with a doctor

3) I thought the doctor would try to persuade me not to use the therapy

4) I didn’t have time to consult with a doctor

5) Other (Please specify: ___________)

Q8. Would you recommend the above therapies to other people?

1) Yes 2) No 3) Not sure

| Information about adverse effects |
| --- |

****** Please answer Q9 **if you selected even one item in “4) Adverse effects” in Q1-7**.

[For Q9, please **refer to the table** when responding. **Please look at the table on the next page** to guide your responses.]

Q9-1. Please select and mark the **symptoms of adverse effects** (duplicate responses are allowed).

**1) Systemic response**

➀ Fever ➁ Reduced appetite ➂ Swelling (systemic oedema) ➃ Fatigue (lethargy) ➄ Weight loss ➅ Weight gain ➆ Flushing

**2) Skin and related tissues**

➀ Hives ➁ Rash ➂ Itching (pruritus) ➃ Erythema ➄ Dermatitis ➅ Skin discoloration ➆ Flaking ➇ Bleeding

➈ Bruising ➉ Burns ⑪ Scarring ⑫ Hair loss ⑬ Vascular oedema ⑭ Cellulitis

**3) Eyes/Nose/Ears/Mouth/Throat**

➀ Dry mouth ➁ Vocal changes ➂ Increased intraocular pressure ➃ Tinnitus

➄ Visual impairment ➅ Auditory impairment ➆ Taste abnormalities

**4) Cardiovascular system**

➀ Chest pain ➁ Tachycardia ➂ Fainting ➃ Hypotension ➄ Hypertension ➅ Arrhythmia

**5) Gastrointestinal system**

➀ Nausea ➁ Vomiting ➂ Heartburn ➃ Dyspepsia ➄ Constipation ➅ Diarrhoea ➆ Abdominal pain ➇ Gastrointestinal bleeding

**6) Liver and gall bladder**

➀ Liver toxicity (elevated AST, ALT) ➁ Elevated bilirubin

**7) Respiratory system**

➀ Coughing ➁ Shortness of breath ➂ Breathing difficulties (bradypnea) ➃ Traumatic pneumothorax

**8) Blood**

➀ Anaemia ➁ Leukopenia ➂ Coagulopathy

**9) Kidneys**

➀ Frequent urination ➁ Hematuria ➂ Proteinuria ➃ Kidney dysfunction

**10) Neuropsychiatric symptoms**

➀ Dizziness (vertigo) ➁ Headache ➂ Anxiety ➃ Sleep disorder ➄ Drowsiness ➅ Depressive mood ➆ Hypersensitivity

➇ Hand/foot tremor ➈ Language disorder ➉ Impaired consciousness ⑪ Hyperactivity ⑫ Delirium

**11) Endocrine-urogenital system**

➀ Dysuria ➁ Lower urinary tract disease ➂ Irregular menstruation ➃ Sexual dysfunction ➄ Reduced libido

**12) Musculoskeletal system**

➀ Joint pain ➁ Muscle pain ➂ Osteoporosis ④ Restricted movement ➄ Other ______________

**13)** **Other** ___________________________

Q9-2. What is the name of the therapy that you suspect of causing the adverse effects?

Q9-3. Please rate the severity of the symptoms you experienced due to the adverse effects?

1) Mild: No need for treatment and not significant impairing normal daily living

2) Moderate: Significant impairment of normal daily living that may require treatment, but recovers after treatment

3) Severe: Severe adverse effects that need a high level of treatment and leave sequelae

Q9-4 Did you experience serious adverse effects?

(Serious adverse effects are defined as the following circumstances:

A life-threatening condition or a condition leading to death / A condition requiring hospitalization or extension of the duration of hospitalization / Continuous or severe disability or dysfunction / Congenital deformity or abnormalities / Other medically important circumstances)

1) Yes 2) No

Q9-5 When did you first develop symptoms of the adverse effects?

Q9-6 When did the symptoms of the adverse effects disappear?

Q9-7 Where there any dose changes in the relevant folk therapy after you developed symptoms of adverse effects?

1) The dose and procedure were maintained. 2) The dose/frequency were reduced.

3) The medication/procedure was stopped.

Q9-8 Did you recover from the symptoms of adverse effects?

1) Yes, I recovered. 2) I am still recovering.

3) I have not recovered. I still have sequelae.

| Adverse effect no. | Q9-1.  Adverse effect symptoms  (Please refer to the type of therapy and mark the relevant number with a √) | Q9-2.  Please provide the name of suspected folk therapy | Q9-3.  Please indicate the severity of the adverse effect symptoms  1) Mild  2) Moderate  3) Severe | Q9-4.  Did you experience severe adverse effects?  (death, life threatening condition, hospitalisation, disability, deformity) | Q9-5  When did you develop symptoms of the adverse effects? | Q9-6  When did the symptoms of the adverse effects disappear? | Q9-7  What changes did you make to the folk therapy?  1) None  2) Reduced dose/frequency  3) Stopped therapy | Q9-8  How have you recovered from the adverse effects?  1) Recovered  2) Still recovering  3) Cannot recover (sequelae) |
| --- | --- | --- | --- | --- | --- | --- | --- | --- |
| 1 | **1. Systemic response**  ➀ Fever  ➁ Reduced appetite  ➂ Systemic oedema  ➃ Fatigue  ➄ Weight loss  ➅ Weight gain  ➆ Flushing |  | 1) 2) 3) | 1) 2) | MM/DD/YYYY | MM/DD/YYYY | 1) 2) 3) | 1) 2) 3) |
| 2 | **2. Skin and related tissues**  ➀ Hives  ➁ Rash  ➂ Itching  ➃ Erythema  ➄ Dermatitis  ➅ Skin discoloration  ➆ Flaking  ➇ Bleeding  ➈ Bruising  ➉ Burns  ⑪ Scarring  ⑫ Hair loss  ⑬ Vascular oedema  ⑭ Infection |  | 1) 2) 3) | 1) 2) | MM/DD/YYYY | MM/DD/YYYY | 1) 2) 3) | 1) 2) 3) |
| 3 | **3. Eyes/Nose/Ears/Mouth/Throat**  ➀Dry mouth ➁Vocal changes  ➂Increased intraocular pressure  ➃Tinnitus  ➄Visual impairment  ➅Auditory impairment  ➆Taste abnormalities |  | 1) 2) 3) | 1) 2) | MM/DD/YYYY | MM/DD/YYYY | 1) 2) 3) | 1) 2) 3) |
| 4 | **4. Cardiovascular system**  ➀ Chest pain  ➁ Tachycardia  ➂ Fainting  ➃ Hypotension  ➄ Hypertension  ➅ Arrhythmia |  | 1) 2) 3) | 1) 2) | MM/DD/YYYY | MM/DD/YYYY | 1) 2) 3) | 1) 2) 3) |
| 5 | **5. Gastrointestinal system**  ➀ Nausea  ➁ Vomiting  ➂ Heartburn  ➃ Dyspepsia  ➄ Constipation  ➅ Diarrhoea  ➆ Abdominal pain  ➇ Gastrointestinal bleeding |  | 1) 2) 3) | 1) 2) | MM/DD/YYYY | MM/DD/YYYY | 1) 2) 3) | 1) 2) 3) |
| 6 | **6. Liver and gall bladder**  ➀Liver toxicity (elevated AST, ALT)  ➁Elevated bilirubin |  | 1) 2) 3) | 1) 2) | MM/DD/YYYY | MM/DD/YYYY | 1) 2) 3) | 1) 2) 3) |
| 7 | **7. Respiratory system**  ➀ Coughing  ➁ Shortness of breath  ➂ Breathing difficulties  ➃ Traumatic pneumothorax |  | 1) 2) 3) | 1) 2) | MM/DD/YYYY | MM/DD/YYYY | 1) 2) 3) | 1) 2) 3) |
| 8 | **8. Blood**  ➀ Anaemia  ➁ Leukopenia  ➂ Coagulopathy |  | 1) 2) 3) | 1) 2) | MM/DD/YYYY | MM/DD/YYYY | 1) 2) 3) | 1) 2) 3) |
| 9 | **9. Kidneys**  ➀ Frequent urination  ➁ Hematuria  ➂ Proteinuria  ➃ Kidney dysfunction |  | 1) 2) 3) | 1) 2) | MM/DD/YYYY | MM/DD/YYYY | 1) 2) 3) | 1) 2) 3) |
| 10 | **10. Neuropsychiatric**  ➀ Dizziness  ➁ Headache  ➂ Anxiety  ➃ Sleep disorder  ➄ Drowsiness  ➅ Depressive mood  ➆ Hypersensitivity  ➇ Hand/foot tremor  ➈ Language disorder  ➉ Impaired consciousness  ⑪ Hyperactivity  ⑫ Hallucinations  ⑬ Anxiety |  | 1) 2) 3) | 1) 2) | MM/DD/YYYY | MM/DD/YYYY | 1) 2) 3) | 1) 2) 3) |
| 11 | **11. Endocrine-urogenital system**  ➀ Dysuria  ➁ Lower urinary tract disease  ➂ Irregular menstruation  ➃ Sexual dysfunction  ➄ Reduced libido |  | 1) 2) 3) | 1) 2) | MM/DD/YYYY | MM/DD/YYYY | 1) 2) 3) | 1) 2) 3) |
| 12 | **12. Musculoskeletal system**  ➀ Joint pain  ➁ Muscle pain  ➂ Osteoporosis  ➃ Restricted movement  ➄ Other _____________ |  | 1) 2) 3) | 1) 2) | MM/DD/YYYY | MM/DD/YYYY | 1) 2) 3) | 1) 2) 3) |
| 13 | **13) Other symptoms** |  | 1) 2) 3) | 1) 2) | MM/DD/YYYY | MM/DD/YYYY | 1) 2) 3) | 1) 2) 3) |

Q10. Did you receive hospital treatment for the above adverse effects?

1) Yes **☞ Q10-1** 2) No

Q10-1. Please describe in detail the dates, types, contents, and findings of examinations.

________________________________________________________________________________________________________________________________________________________________________________________________________________

| Other |
| --- |

Q11. Please freely describe any desires you have regarding national or health authority policies for rare and intractable diseases (including ALS and muscle dystrophy).

________________________________________________________________________________________________________________________________________________________________________________________

____________________________________________________________________________________________

____________________________________________________________________________________________

____________________________________________________________________________________________

____________________________________________________________________________________________

____________________________________________________________________________________________

____________________________________________________________________________________________

| **** This will be filled in by the investigator. Respondents should not write anything below.** | | | |
| --- | --- | --- | --- |
| **Date of validation** | HH: MM, MM/ DD/2013 | **Validation results** |  |

*** Thank you very much for taking the time to respond. ***
