# Supplementary material for: Complementary and Alternative Medicine Use in Amyotrophic Lateral Sclerosis Cases in South Korea
Source: Evid Based Complement Alternat Med. 2019 Jul 25;2019:4217057. doi: 10.1155/2019/4217057 (PMC6683772; doi:10.1155/2019/4217057)
Supplement: Supplementary Materials — Supplementary Appendix 1: a questionnaire survey conducted to understand the treatment behaviours relating to ALS and muscle dystrophy in ALS patients. Based on the results of this survey, we aimed to ascertain the precise state of methods used by ALS patients in Korea to treat the disease and improve health, hoping that data will be used to establish policies that improve treatments for ALS patients in Korea. Supplementary Appendix 2: prevalence of CAM use by CAM type (n = 195). Supplementary Appendix 3: used modalities in dietary treatments (n = 46). [file 4217057.f1.zip › 4217057.f1/Supplemental Appendix 2-3.docx]

**Supplemental Appendix 2. Prevalence of CAM use by CAM type (n = 195)**

| **CAM type** | **Prevalence** | |
| --- | --- | --- |
|  | **n** | **(%)** |
| **Natural products** | **91** | **(46.7)** |
| Dietary treatment * | 46 | (23.6) |
| Other dietary therapies † | 4 | (2.1) |
| Herbal medicines prescribed by non-institutional practitioners | 1 | (0.5) |
| Functional food | 38 | (19.5) |
| Herbal medicine-based products | 17 | (8.7) |
| Other functional foods § | 21 | (10.8) |
| External application of natural product | 2 | (1.0) |
| **Mind and body medicine** | **98** | **(50.3)** |
| Acupuncture | 14 | (7.2) |
| Moxibustion | 10 | (5.1) |
| Cupping therapy | 6 | (3.1) |
| Bloodletting therapy | 6 | (3.1) |
| Chuna manipulative treatment | 1 | (0.5) |
| Chiropractic treatment | 2 | (1.0) |
| Massage | 19 | (9.7) |
| Thermotherapy | 13 | (6.7) |
| Special exercise therapy | 2 | (1.0) |
| Physical therapy with home medical devices | 11 | (5.6) |
| External qi treatment | 5 | (2.6) |
| Qigong training | 3 | (1.5) |
| Spiritual treatment | 5 | (2.6) |
| Activity therapy | 1 | (0.5) |
| **Others** | **6** | **(3.1)** |

^*^ Includes vegetable and fruit juices, mushroom, greens and seaweed, charcoal and bamboo salt, animal products, medicinal tea, medicinal liquor, etc.

† Includes specific dietary treatment according to the yin-yang 5-element theory, etc.

^§^ Includes vitamins, omega-3, saw palmetto, etc.

CAM, complementary and alternative medicine

**Supplemental Appendix 3. Used Modalities in Dietary treatments (n = 46)**

| **CAM type** | **Prevalence** | |
| --- | --- | --- |
|  | **n** | **(%)** |
| **Dietary treatment** | 46 | (100) |
| Vegetable juices^*^ | 3 | (6.5) |
| Fruit juices^†^ | 19 | (41.3) |
| Mushroom^‡^ | 5 | (10.9) |
| Bamboo salt | 3 | (6.5) |
| Animal products^§^ | 10 | (21.7) |
| Medicinal tea ^‖^ | 4 | (8.7) |
| Medicinal liquor^¶^ | 2 | (4.3) |

* Includes yam, fleeceflower, and [vegetable sponge](https://endic.naver.com/enkrEntry.nhn?entryId=8f767fc0f64f4ebca7599cce80c87012&query=%EC%88%98%EC%84%B8%EB%AF%B8)

† Includes grape juice, pear juice, Korean blackberry juice, strawberry, cherry tomato, juice with pear and black garlic, and mixed juice with broccoli, banana, and tomato

‡ Includes Sanghwang mushrooms (Phellinus linteus), Shiitake mushrooms, Lingzhi mushrooms, Chaga mushrooms, oyster mushroom, [Hericium erinaceum](https://endic.naver.com/enkrEntry.nhn?entryId=eb8e114b042b452e9411efc14af04923&query=%EB%85%B8%EB%A3%A8%ED%92%8D%EB%8E%85%EC%9D%B4+%EB%B2%84%EC%84%AF)

§ Includes Dog elixir, Chicken feet, Duck, Freshwater eel, Mudfish, Beef, Pollack,[Cow Bone](https://endic.naver.com/enkrEntry.nhn?entryId=2fc15ed22f2548c1b851c7fff1d87b3f&query=%EC%86%8C%EB%BC%88), and Dog

‖ Includes cassia seed, Bamboo leaf, Omija

¶ Includes Medicinal liquor made by Fleeceflower, and Medicinal liquor made by black soybean
